# Kruppel homolog 1 modulates ROS production and antimicrobial peptides expression in shrimp hemocytes during infection by the *Vibrio parahaemolyticus* strain that causes AHPND

**DOI:** 10.3389/fimmu.2023.1246181

**Published:** 2023-08-29

**Authors:** Zhou Zheng, Shangjie Liu, Zhongyang Lin, Jude Juventus Aweya, Zhihong Zheng, Yongzhen Zhao, Xiuli Chen, Shengkang Li, Yueling Zhang

**Affiliations:** ^1^ Institute of Marine Sciences and Guangdong Provincial Key Laboratory of Marine Biotechnology, Shantou University, Shantou, China; ^2^ Department of Medical Laboratory and Department of Reproductive Medicine, Luohu Clinical College of Shantou University Medical College, Shantou University, Shantou, China; ^3^ College of Ocean Food and Biological Engineering, Fujian Provincial Key Laboratory of Food Microbiology and Enzyme Engineering, Jimei University, Xiamen, Fujian, China; ^4^ Guangxi Academy of Fishery Sciences, Guangxi Key Laboratory of Aquatic Genetic Breeding and Healthy Aquaculture, Nanning, China

**Keywords:** *Penaeus vannamei*, AHPND, Kr-h1, IMD/Relish, antimicrobial peptides, ROS

## Abstract

Shrimp aquaculture has been seriously affected by acute hepatopancreatic necrosis disease (AHPND), caused by a strain of *Vibrio parahaemolyticus* that carries the Pir toxin plasmids (*V. parahaemolyticus*_(AHPND)_). In this study, the transcription factor, Kruppel homolog 1-like of *Peneaus vannamei* (*Pv*Kr-h1), was significantly induced in shrimp hemocytes after *V. parahaemolyticus*_(AHPND)_ challenge, suggesting that *Pv*Kr-h1 is involved in shrimp immune response. Knockdown of *Pv*Kr-h1 followed by *V. parahaemolyticus*_(AHPND)_ challenge increased bacterial abundance in shrimp hemolymph coupled with high shrimp mortality. Moreover, transcriptome and immunofluorescence analyses revealed that *Pv*Kr-h1 silencing followed by *V. parahaemolyticus*_(AHPND)_ challenge dysregulated the expression of several antioxidant-related enzyme genes, such as Cu-Zu SOD, GPX, and GST, and antimicrobial peptide genes, i.e., CRUs and PENs, and reduced ROS activity and nuclear translocation of Relish. These data reveal that *Pv*Kr-h1 regulates shrimps’ immune response to *V. parahaemolyticus*_(AHPND)_ infection by suppressing antioxidant-related enzymes, enhancing ROS production and promoting nuclei import of *Pv*Relish to stimulate antimicrobial peptide genes expression.

## 1 Introduction

Acute hepatopancreatic necrosis disease (AHPND), caused by a strain of *Vibrio parahaemolyticus* that contains a plasmid encoding the binary Photorhabdus insect-related (Pir) toxin genes PirA/PirB (1), is a highly pathogenic and transmissible disease in aquaculture (2). This disease was first reported in 2009 in China and other countries (3). The rapid onset and high mortality associated with AHPND, especially among shrimps (i.e., *Penaeus vannamei*, *Penaeus monodon*, *Macrobrachium rosenbergii*, *Fenneropenaeus chinensis*, etc.), results in huge economic losses to shrimp aquaculture (4).

Although AHPND was identified over two decades ago, the immune response mechanisms and the disease pathogenesis remain unclear. It has been shown in shrimp that *V. parahaemolyticus*_(AHPND)_ infection stimulates innate immune defense mechanisms *via* the Toll-like receptor (TLR) and immune deficiency (IMD) signaling pathways (5), expression of lectins (6), and phenoloxidases (7) to prevent bacteria spread, and induces hemocytes phagocytosis and apoptosis of bacteria (8, 9). Many transcription factors (TFs), such as Dorsal (10), Relish (11), signal transducer and activator of transcription (STAT) (12), activating transcription factor 6 (ATF6) (13), AP-1 (c-Jun and c-Fos) (14), sterol regulatory element binding protein (SREBP) (15), CSL (CBF-1/RBP-J kappa -, and suppressor of hairless (Su (H)) or Lag-1) (16), have been implicated in shrimp antimicrobial immunity *via* immune-related genes or directly in response to pathogens or indirectly by modulating antimicrobial peptides (AMPs) genes. In arthropods, such as insects, the transcription factor Kruppel homolog 1 (Kr-h1) plays crucial defense functions (17) but the role of Kr-h1 in shrimp immune defense is unknown, even though it is induced in shrimp hemocytes during *V. parahaemolyticus*_(AHPND)_ challenge (9).

Kruppel homolog 1 (Kr-h1) was first identified in the fruitfly *Drosophila melanogaster* (18) and recently in some crustaceans, such as the Chinese mitten crab *Eriocheir sinensis* and the swimming crab *Portunus trituberculatus* (19, 20). Structurally, Kr-h1 is similar to other Kruppel family members and contain zinc finger domains (19, 20), that is mainly regulated by the juvenile hormone (JH) and ecdysone and have been implicated in the regulation of metamorphosis in insects (21). A recent transcriptome study of the red flour beetle *Tribolium castaneum* has shown that Kr-h1 responds to immune challenges (22). Similarly, transcriptome analysis of the pacific white shrimp hemocytes challenged with *V. parahaemolyticus*_(AHPND)_ has revealed a significant dysregulation of Kr-h1, suggesting that the of Kr-h1 homolog in *P. vannamei* (*Pv*Kr-h1) could be involved in antibacterial immune response in penaeid shrimps. Here, we explored how *Pv*Kr-h1 modulates shrimp immune response during *V. parahaemolyticus*_(AHPND)_ infection and the molecular mechanisms involved.

## 2 Materials and methods

### 2.1 Screening of previously published transcriptome data

The DESeq2 online software (Version 1.24.0, http://bioconductor.org/packages/stats/bioc/DESeq2/) was used to screen for the differentially expressed genes (DEGs) in our previously published transcriptome data (GenBank accession number: PRJNA385392). This transcriptome data was generated from hemocytes samples collected from shrimp infected with two strains of *V. parahaemolyticus*, i.e., non-AHPND-causing strain [MCCC1H00058, purchased from Marine Culture Collection of China (MCCC)] and AHPND-causing strain [a gift from Professor Lo Chufang (National Cheng Kung University, Taiwan, China)] (9, 23). A fold change (FC) >= 2 and false discovery rate (FDR) < 0.01 were used for screening the DEGs, where FC represents the ratio of expression of the same gene in two samples, while FDR was obtained by correcting the *p-value* for the significant difference. The screened DEGs were categorized into two sets, i.e., *V. parahaemolyticus*_(AHPND)_ vs. control and *V. parahaemolyticus* vs. control, designated as Gene Set A1 after removing redundancy. Next, all the predicted transcription factor sequences from the AnimalTFDB (Version 3.0, http://bioinfo.life.hust.edu.cn/AnimalTFDB/#!/) were downloaded in Fasta format and designated as TF-data (24). Finally, Gene Set A1 and TF-data genes were compared using local BLAST+ software (Version 2.9.0, ftp://ftp.ncbi.nlm.nih.gov/blast/executables/blast+/2.9.0/) to identify differentially expressed transcription factors (25).

### 2.2 Experimental animals, tissue collection, RNA extraction, cDNA synthesis, and RT-qPCR analysis

Healthy shrimp *P. vannamei* (weight of 8 ± 2 g each), bought from a local shrimp farm, Shantou Huaxun Aquatic Product Corporation (Shantou, Guangdong, China), were cultured in aerated laboratory tanks containing seawater (1% salinity) at 25°C for 3-5 days laboratory acclimatization before experiments. All animal experiments were performed according to the guidelines and approved by the Animal Research and Ethics Committees of Shantou University, Guangdong, China. Hemolymph was collected into an equal volume of precooled acid citrate dextrose (ACD) anti-coagulant buffer (27 mM C_6_H_5_O_7_Na_3_·2 H_2_O, 33 mM C_6_H_8_O_7_, 110 mM C_6_H_12_O_6_, 140 mM NaCl, pH 6.0) and the hemocytes harvested by centrifugation at 500 g for 10 min at 4°C. Other tissues (i.e., muscle, intestine, gill, hepatopancreas, nerve, heart, stomach, and eyestalk), were collected and processed as previously described (26). Total RNA was extracted from the tissues using the RNAFAST 200 kit (FeiJie, Shanghai, China) following the manufacturer’s instructions. The RNA concentration was determined with a NanoDrop2000 spectrophotometer (Nano-drop Technologies, Wilmington, DE), and the RNA quality was ascertained using the A260/280 ratio (>= 2.0) and on 1% agarose gel electrophoresis. Only good-quality RNA samples were used for further analysis. First-strand cDNA synthesis was carried out with 1.0 μg of total RNA using the EasyScrpt One-Step gDNA Removal and cDNA Synthesis SuperMix kit (TransGen Biotech, Beijing, China), following the manufacturer’s instructions.

RT-qPCR was performed using Master SYBR Green I system (GenStar, Beijing, China) on the Roche Light-Cycler 480 system (Roche, Switzerland) using the following thermal cycling parameters: one cycle at 95°C for 10 min, followed by 40 cycles at 95°C for 15 s and 60°C for 30 s. All the gene-specific primers were listed in **Table 1**, the elongation factor 1 alpha gene from *P. vannamei* (*Pv*EF1α) was used as an internal control. The relative gene expression was calculated using the 2-^ΔΔCT^ method. Samples were analyzed in triplicates using five shrimps per group.

**Table 1:** Sequence of primers used in this article.

Primer	Sequence (5’-3’)
PCR	
F-Kr-h1	tccaggggcccctgggatccATGGCCATGATGCCCCAGGGAA
R-Kr-h1	cccgggaattccggggatccCTAGCAGTAGTGAAGGAACTCG
dsRNA	
dsRNA-Kr-h1-T7F	GGATCCTAATACGACTCACTATAGGCAGCTAATGGTCGGGTCG
dsRNA-Kr-h1-T7R	GGATCCTAATACGACTCACTATAGGCGTGGTTGTAGCCAAAGG
dsRNA-Kr-h1-F	CAGCTAATGGTCGGGTCG
dsRNA-Kr-h1-R	CGTGGTTGTAGCCAAAGG
dsRNA-EGFP-T7F	GGATCCTAATACGACTCACTATAGGCGTAAACGGCCACAAGTT
dsRNA-EGFP-T7R	GGATCCTAATACGACTCACTATAGGTTCACCTTGATGCCGTTC
dsRNA-EGFP-F	CGTAAACGGCCACAAGTT
dsRNA-EGFP-R	TTCACCTTGATGCCGTTC
RT-qPCR	
qF-LOC113823218	TCGCTACTGGGCTCTTGC
qR-LOC113823218	GACCACGCTGTGAACCTGA
qF-LOC113809796	CAGTACCGCAAGGTGATGAA
qR-LOC113809796	GAGCTTCTTCAGGTGGGTCA
qF- LOC113822590	TGGCCGTCACCGCCTAC
qR- LOC113822590	CGCCGGACCGCTTCTT
qF-Kr-h1	TGGAACAAAGAATGGCTGAAC
qR-Kr-h1	CGACGGGTATGTGAGGGTG
qF-EF-1α	TATGCTCCTTTTGGACGTTTTGC
qR-EF-1α	CCTTTTCTGCGGCCTTGGTAG
qF-PirB	GTGGGCTGATAACGACTC
qR-PirB	ACCAACAGCAGGTGAATA
qF-CRU1	GCCCACGAACCAGAGACAC
qR-CRU1	CCTGCGATCCGAAGAATGA
qF-CRU2	TCGCTTAGGAGGAGGATTCG
qR-CRU2	TAATTGCAGTTGAATCCGCCT
qF-CRU3	TCTGGTCGTGTTGGTCTTGGT
qR-CRU3	GACGTCGCTCGTATCTGGG
qF-PEN2	ACCACCGTTCAGACCTGTTTG
qR-PEN2	TTCTCCGTCAATTTCTTTATCCTTT
qF-PEN3	GCCTATTGGTCCATACAACGG
qR-PEN3	GTTTTCATCGTGTTCTCCGTCA
qF-PEN4	GCCCGTTACCCAAACCATC
qR-PEN4	TCTTCTCCATCAACCAGACTATCC

### 2.3 Pathogen challenge, *in vivo* gene silencing, and shrimp mortality analysis

To examine the response of *Pv*Kr-h1 to immune challenge, healthy shrimp were randomly divided into four groups, with 100 shrimps per group, before being intramuscularly injected *via* the third abdominal segment with *V. parahaemolyticus*_(AHPND)_ (1 × 10^5^ CFU), *V. parahaemolyticus* (1 × 10^5^ CFU), *Streptococcus iniae* (1 × 10^5^ CFU) or an equal volume of sterile 0.01 M PBS (137 mM NaCl, 8 mM Na_2_HPO_4_, 2 mM NaH_2_PO_4_, pH 7.4), respectively. Hemocytes were collected from five randomly selected shrimps per group at 0, 12, 24, 48, and 72 h post-injection (hpi). Total RNA extraction, cDNA synthesis and RT-qPCR analysis were conducted as described in subsection 2.2.

*In vivo* knockdown of *Pv*Kr-h1 was performed using double-stranded RNA (dsRNA) that targets a portion of the ORF sequence of *Pv*Kr-h1 and the EGFP gene as a control. The specific primers (**Table 1**) used to synthesize the dsRNA were designed with Primer Premier 5 and synthesized using the HiScribe™T7 Quick High Yield RNA Synthesis Kit (New England Biolabs, Ipswich, MA) following the manufacturer’s protocol. For the knockdown experiments, 300 healthy shrimp were randomly divided into two groups (experimental and control groups), with the experimental group shrimp each intramuscularly injected *via* the third abdominal segment with 10 μg ds*Pv*Kr-h1, whereas the control group shrimp were each injected with an equal amount of dsEGFP. Hemocytes were collected from five randomly selected shrimp per group at 24, 48, and 72 h post-injection for total RNA extraction, cDNA synthesis, and RT-qPCR analysis to determine knockdown efficiency. To determine shrimp mortality rate after *Pv*Kr-h1 knockdown followed by pathogen challenge, shrimp injected with ds*Pv*Kr-h1 (experimental group) and dsEGFP (control group) for 48 h were then further divided into three subgroups (35 shrimp each), and injected with *V. parahaemolyticus*_(AHPND)_ (1 × 10^5^ CFU), *V. parahaemolyticus* (1 × 10^5^ CFU) or an equal volume of sterile 0.01 M PBS, respectively. Shrimp mortality was recorded at 0, 12, 18, 24, 36, 48, 60, and 72 h post-bacteria challenge, and the cumulative mortality rate was calculated. The mortality graph was plotted using GraphPad Prism 8 software. Statistical significance was determined by Kaplan-Meier survival analysis, and the p-values indicated.

### 2.4 Genomic DNA extraction and bacterial load analysis

Healthy shrimps were randomly divided into two groups, with one group injected with 10 μg of ds*Pv*Kr-h1 while the other group was injected with an equal amount of dsEGFP. At 48 h post-injection, shrimps from each group were further divided into two subgroups, with each subgroup injected with *V. parahaemolyticus*_(AHPND)_ (1 × 10^5^ CFU) or with an equal volume of sterile 0.01 M PBS. At 72 h post the first injection, hemolymph was collected from five shrimp per group and centrifuged at 500g for 10 min at 4°C to collect the supernatant. The supernatant was further centrifuged at 16,000 g for 10 min at 4°C to collect the pellet followed by aseptic extraction of genomic DNA (gDNA) using the TIANamp Marine Animals DNA Kit (TIANGEN, Beijing, China) following the manufacturer’s protocols. To identify bacterial (*V. parahaemolyticus*_(AHPND)_) abundance in the hemolymph samples, RT-qPCR-based methods were used. First, a standard curve was prepared using *V. parahaemolyticus*_(AHPND)_ strains and gene-specific primers (PirB, **Table 1**) as previously described (27). Next, RT-qPCR assay was performed using primers specific to the *V. parahaemolyticus*_(AHPND)_ strain (PirB, **Table 1**) and the DNA extracted above. Finally, the RT-qPCR results and the prepared standard curve were used to calculate the bacterial abundance.

### 2.5 Sample preparation and transcriptome profiling

Hemocytes collected from shrimp injected with ds*Pv*Kr-h1 or dsEGFP followed by injection with *V. parahaemolyticus*_(AHPND)_ or PBS (i.e., ds*Pv*Kr-h1 + *V. parahaemolyticus*_(AHPND)_, dsEGFP + *V. parahaemolyticus*_(AHPND)_, ds*Pv*Kr-h1 + PBS, or dsEGFP + PBS) as described in subsection 2.4 were used for total RNA extracted using the TRIzol Plus (Invitrogen, Carlsbad, CA) following the manufacturer’s protocols. Next, the high-quality RNA samples were used to construct cDNA libraries followed by RNA sequencing on the Illumina Novaseq 6000 platform by a commercial company (Majorbio Biotech, Shanghai, China). The data obtained was then analyzed as described previously (9). The data has been submitted to GenBank under accession number PRJNA875156. After the RNAseq data analysis, the significant DEGs in the various groups (i.e., ds*Pv*Kr-h1 + *V. parahaemolyticus*_(AHPND)_ vs dsEGFP + *V. parahaemolyticus*_(AHPND)_ and ds*Pv*Kr-h1 + PBS vs dsEGFP + PBS) were selected for functional annotation using Kyoto encyclopedia of genes and genomes (KEGG) pathway enrichment analysis on the KEGG database (http://www.genome.jp/kegg/) as described previously (9). Based on the functional annotation results and previous studies on shrimp, key immune-related genes were chosen from the DEGs in group ds*Pv*Kr-h1 + *V. parahaemolyticus*_(AHPND)_ vs dsEGFP + *V. parahaemolyticus*_(AHPND)_. These DEGs were then validated using RT-qPCR with gene-specific primers (**Table 1**) as described in subsection 2.2.

### 2.6 Analysis of ROS levels in hemocytes

ROS levels in hemocytes were determined using the Reactive Oxygen Species Assay Kit (Beyotime biotechnology, Shanghai, China) which uses fluorescent compound, DCFH-DA, to measure hydroxyl, peroxyl, and other ROS activity in the cell. Briefly, freshly isolated hemocytes from five shrimp were incubated with 10mM DCFH-DA in PBS for 20 min at 37°C (1 × 10^6^ cells were used per group). Next, cells were harvested by centrifugation and suspended in PBS, then and measured at 485-nm (excitation) and 527-nm (emission) wavelengths on a microplate reader (Synergy HTX; BioTek, Winooski,VT). The level of ROS was calculated as fluorescence intensity. Triplicate samples were measured per treatment for at least three independent experiments. Statistical significance was determined using the Student’s t-test relative to control.

### 2.7 Immunofluorescence assay

Immunofluorescence analysis was used to analyze the translocation of *Pv*Relish into the nucleus as described previously (28). Briefly, hemocytes were collected from shrimp injected with ds*Pv*Kr-h1 or dsEGFP followed by injection with *V. parahaemolyticus*_(AHPND)_ or PBS (i.e., ds*Pv*Kr-h1 + *V. parahaemolyticus*_(AHPND)_, dsEGFP + *V. parahaemolyticus*_(AHPND)_, ds*Pv*Kr-h1 + PBS, and dsEGFP + PBS) as described in subsection 2.5 The collected cells were then resuspended in Insect-XPRESS™ media (LONZA, Basel, Switzerland) followed by seeding 2×10^6^ cells per well onto glass-bottom plates, before being incubated at 28°C for 2 h. Next, cells were fixed with 4% paraformaldehyde at room temperature for 15 min before being washed three times with 0.01 M PBS, followed by incubation with 0.5% Triton X-100 in 0.01 M PBS at room temperature for 20 min. Samples were then washed and incubated with 5% BSA (bovine serum albumin) for 30 min, and then with rabbit anti-*Pv*Relish antisera (1:100, prepared inhouse) overnight at 4°C. After being washed three times with PBST (137 mM NaCl, 8 mM Na_2_HPO_4,_ 2 mM NaH_2_PO_4_, 0.5% Tween-20, pH 7.4), samples were incubated with goat anti-rabbit IgG (H+L) cross-adsorbed secondary antibody (Invitrogen, Carlsbad, CA, 1:1000) for 1 h at room temperature in the dark. Samples were washed three times with PBST, stained with Hochest 33342 (Beyotime, Shanghai, China) for 10 min at room temperature protected from light, followed by washing six times, before being examined under a Confocal Microscope LSM800 (Carl ZEISS, Heidenheim, Germany).

## 3 Results

### 3.1 *Pv*Kr-h1 is induced in shrimp hemocytes upon *V. parahaemolyticus*_(AHPND)_ challenge

**Figure 1:**
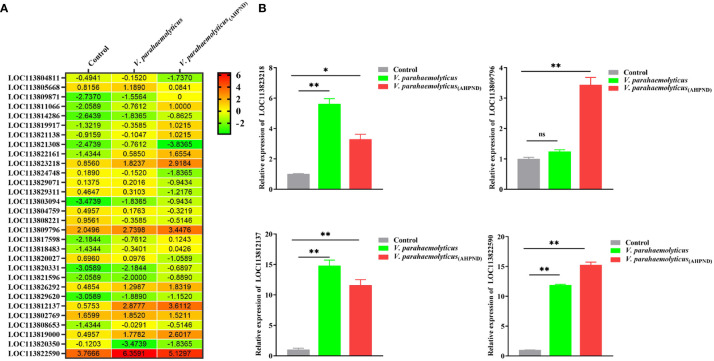
Screening of transcription factors in *P. vannamei* hemocytes after challenge with the AHPND strain of *Vibrio parahaemolyticus* (*V. parahaemolyticus*_(AHPND)_). **(A)** Heatmap showing differentially expressed transcription factors in shrimp hemocytes (data culled from GenBank PRJNA385392). **(B)** Transcript levels of selected transcription factors (LOC113823218, LOC113809796, LOC113812137, and LOC113819000) in hemocytes from *V. parahaemolyticus* and *V. parahaemolyticus*_(AHPND)_ challenged shrimps. The LOC113822590 gene was annotated as a Kruppel homolog 1-like in *P. vannamei* (*Pv*Kr-h1). Statistical analysis was performed by the unpaired Student’s t-test (ns, not significant; *p < 0.05; **p < 0.01).

**Figure 2:**
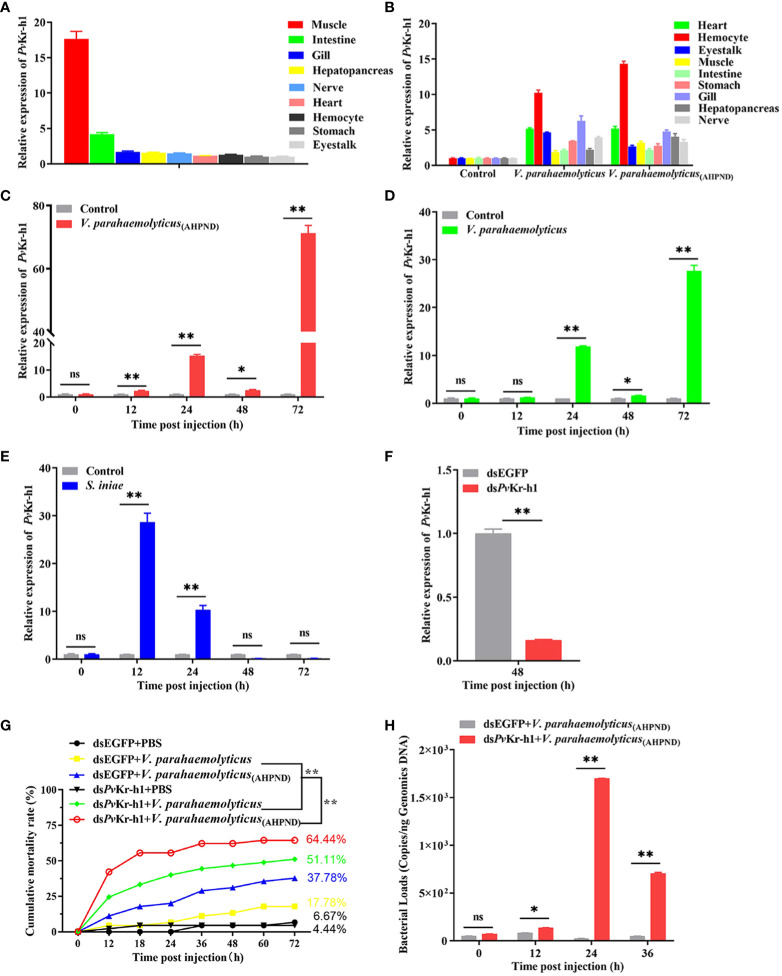
Expression of Kr-h1 in *P. vannamei* in response to immune challenge. **(A)** *Pv*Kr-h1 mRNA levels in healthy shrimp determined by RT-qPCR and normalized to the *Pv*EF-1α gene (internal control). The *Pv*Kr-h1 expression in other tissues was normalized to the tissue with the lowest expression level (eyestalk). **(B)** *Pv*Kr-h1 mRNA expression in different shrimp tissues after challenge with *V. parahaemolyticus*_(AHPND)_, *V. parahaemolyticus*, or PBS (negative control) at 24 h post-injection. **(C-E)** *Pv*Kr-h1 mRNA levels in shrimp hemocytes after challenge with **(C)** *V. parahaemolyticus*_(AHPND)_ **(D)** *V. parahaemolyticus* and **(E)** *S. iniae.* **(F)** Knockdown efficiency of *Pv*Kr-h1 after shrimp were injected with ds*Pv*Kr-h1 or dsEGFP (control) for 48 h Transcript levels were determined by RT-qPCR and normalized to the *Pv*EF-1α gene (internal control). The expression levels of *Pv*Kr-h1 for control groups (dsEGFP or PBS) at each time point were set to 1.0. Data represents mean ± SD (n = 5) for three independent experiments. **(G)** Shrimp mortality (n = 35 per group) was determined after intramuscular injection of shrimp with ds*Pv*Kr-h1 or dsEGFP followed by *V. parahaemolyticus*_(AHPND)_, *V. parahaemolyticus*, or PBS (negative control) and the number of dead shrimps counted and recorded at the indicated time points. **(H)** Bacteria abundance in shrimp hemolymph after *Pv*Kr-h1 knockdown followed by *V. parahaemolyticus*_(AHPND)_ infection. Statistical difference was analyzed by the Student’s t-test and significance was considered at *p <* 0.05 (ns, not significant; *p < 0.05; **p < 0.01).

Screening of our previously published shrimp hemocytes RNA-Seq data (PRJNA385392) revealed the dysregulation of thirty putative transcription factor (TF) genes, among which LOC113823218, LOC113809796, LOC113812137, and LOC113822590 were highly expressed (**Figure 1A**). Validation of the expression of these four genes using RT-qPCR after the infection of shrimps with *V. parahaemolyticus* and *V. parahaemolyticus*_(AHPND)_ (**Figure 1B**) revealed LOC113822590 as the most upregulated gene (**Figure 1B**). LOC113822590 was identified as the *P. vannamei* homolog of the Kruppel homolog 1-like gene (*Pv*Kr-h1) after cloning and sequence alignment (**Figure S1**). Tissue profiling of *Pv*Kr-h1 mRNA transcripts revealed that it was constitutively expressed in all tested tissues i.e., intestine, stomach, eyestalk, heart, hepatopancreas, hemocytes, nerve, gill, and muscle (**Figure 2A**), with muscle expressing the highest. When shrimp were challenged with *V. parahaemolyticus* and *V. parahaemolyticus*_(AHPND),_ mRNA transcript levels were significantly induced in shrimp hemocytes (**Figure 2B**); hence hemocytes were used in all subsequent experiments.

**Figure 3:**
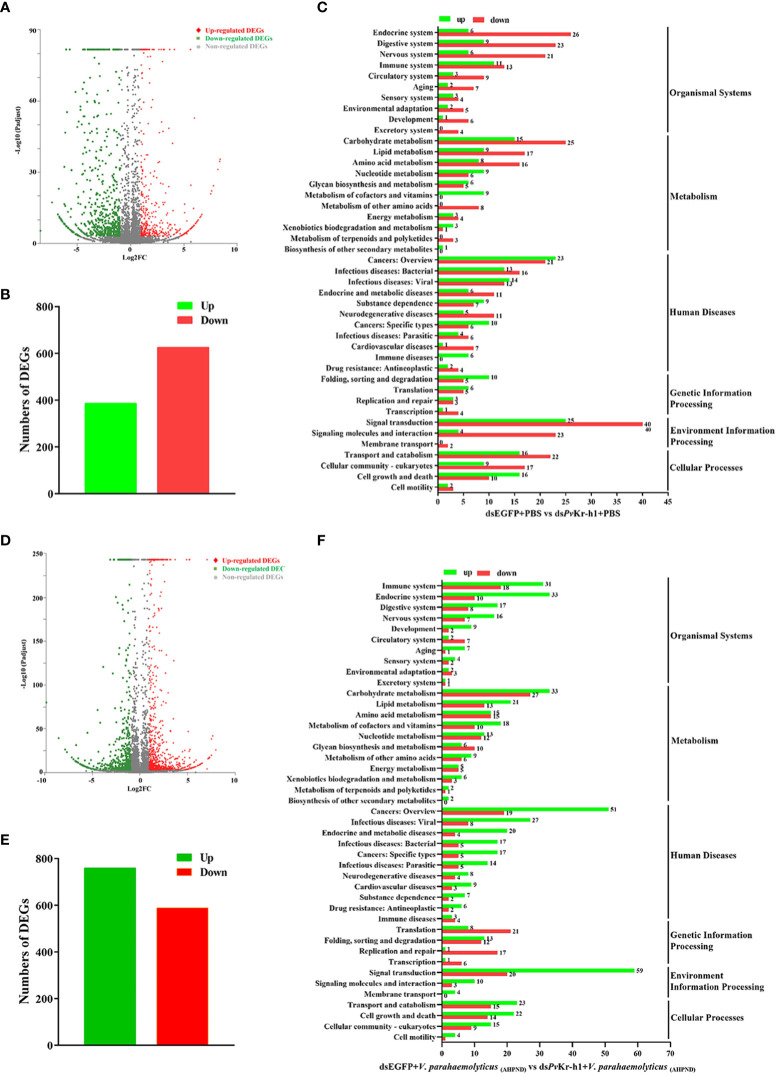
Distribution of differentially expressed genes after *Pv*Kr-h1 knockdown followed by *V. parahaemolyticus*_(AHPND)_ infection. **(A–C)** Differentially expressed genes (DEGs) after shrimp were injected with ds*Pv*Kr-h1 or dsEGFP (control) followed by PBS injection, **(A)** volcano plot, **(B)** bar chart, and **(C)** KEGG pathway enrichment. **(D–F)** DEGs after shrimp were injected with ds*Pv*Kr-h1 or dsEGFP (control) followed by *V. parahaemolyticus*_(AHPND)_ injection, **(D)** volcano plot, **(E)** bar chart, and **(F)** KEGG pathway enrichment.

The mRNA transcript levels were not only induced in hemocytes by Gram-negative (*V. parahaemolyticus* and *V. parahaemolyticus*_(AHPND)_) bacteria (**Figures 2C, D**) but also by Gram-positive (*S. iniae*) bacteria at different time points (**Figure 2E**). For instance, after challenging with *V. parahaemolyticus*_(AHPND)_, the mRNA transcript levels of *Pv*Kr-h1 increased steadily from 12 h and peaked at 72 h (**Figure 2C**). A similar significant increase in *Pv*Kr-h1 expression was observed upon challenge with *V. parahaemolyticus*, with peak expression also found at 72 h (**Figure 2D**). The mRNA transcript levels of *Pv*Kr-h1 after *S. iniae* challenge, significantly at 12 h followed by a gradual decreased to baseline at 48 h (**Figure 3E**). Thus, the three bacteria pathogens could induce *Pv*Kr-h1 expression in shrimp hemocyates, with *V. parahaemolyticus*_(AHPND)_ inducing the strongest.

### 3.2 *Pv*Kr-h1 plays a vital role in shrimp antibacterial immunity

To further explore the role of Kr-h1 in shrimp response to *V. parahaemolyticus*_(AHPND)_ infection, shrimp were depleted of Kr-h1 followed by *V. parahaemolyticus*_(AHPND)_ challenge. We observed that after *Pv*Kr-h1 knockdown (**Figure 2F**) followed by challenge with *V. parahaemolyticus*, *V. parahaemolyticus*_(AHPND)_, or PBS as control, shrimp mortality was significantly higher (*p<0.01*) in the Kr-h1 depleted groups (i.e., ds*Pv*Kr-h1 + *V. parahaemolyticus* or ds*Pv*Kr-h1 + *V. parahaemolyticus*_(AHPND)_) compared with the control groups (i.e., dsEGFP + *V. parahaemolyticus* or dsEGFP + *V. parahaemolyticus*_(AHPND)_) (**Figure 2G**). When hemolymph total bacterial abundance was analyzed before and after *Pv*Kr-h1 knockdown with *V. parahaemolyticus*_(AHPND)_ challenge, a significant increase in total bacteria abundance was observed at 12 hpi (*p<0.05*), 24 hpi (*p<0.01*), and 36 hpi (*p<0.01*) in the knockdown challenged group (ds*Pv*Kr-h1 + *V. parahaemolyticus*_(AHPND)_) compared with the control challenged group (**Figure 2H**). These results further indicate the role of *Pv*Kr-h1 in shrimp antimicrobial response to AHPND.

### 3.3 Transcriptome analysis reveals that *Pv*Kr-h1 modulates shrimp immunity

To further explore how *Pv*Kr-h1 modulates shrimp immune response to *V. parahaemolyticus*_(AHPND)_ infection, we performed RNAseq using shrimp hemocytes after *Pv*Kr-h1 knockdown with PBS challenge. A total of 1014 differentially expressed genes (DEGs) were identified, among which 387 were upregulated while 627 were downregulated (**Figures 3A, B**). Further analysis of the DEGs using KEGG pathway enrichment analysis revealed that the significantly enriched KEGG pathways include signal transduction, cancers-related, carbohydrate metabolism, transport and catabolism, endocrine system, and digestive system (**Figure 3C**). Signal transduction category was the most enriched with 25 upregulated and 40 downregulated genes. Besides, 24 DEGs were enriched in the immune system category (**Figure 3C**).

When the gene expression pattern in the *Pv*Kr-h1 knockdown followed by *V. parahaemolyticus*_(AHPND)_ challenge group was compared with the control group (i.e., dsEGFP + *V. parahaemolyticus*_(AHPND)_), 1349 DEGs were identified, out of which 760 were upregulated and 589 downregulated (**Figures 3D, E**). KEGG analysis of the 1349 DEGs revealed that these they were enriched in several categories, including immune system, cell growth and death, lipid metabolism, amino acid metabolism and signal transduction (**Figure 3F**). Among the DEGs enriched in the immune system category (**Tables S1**, **S2**), antioxidant-related enzymes, such as Copper-zinc superoxide dismutase (Cu-Zn SOD), glutathione peroxidase (GPX), and glutathione-S-transferase (GST), were significantly upregulated, while antimicrobial peptide genes, such as crustins (CRUs) and penaeidins (PENs) were significantly downregulated.

### 3.4 *Pv*Kr-h1 mediates ROS production in shrimp antimicrobial response to *V. parahaemolyticus*_(AHPND)_ challenge

**Figure 4:**
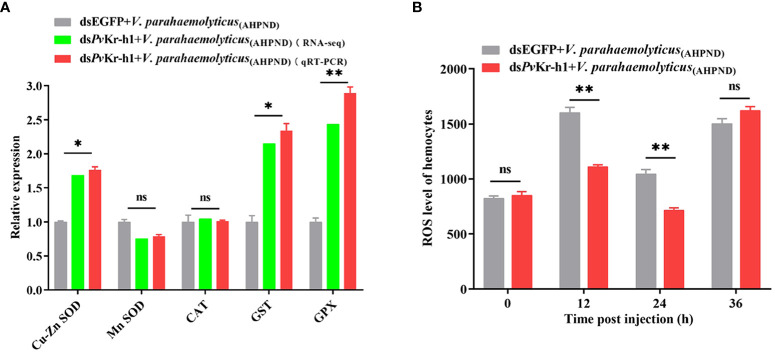
*Pv*Kr-h1 plays an important role in *V. parahaemolyticus*_(AHPND)_-induced hemocytes ROS production. **(A)**. RT-qPCR and RNA-seq analysis of antioxidant-related genes after shrimp were injected with ds*Pv*Kr-h1 or dsEGFP (control) followed by *V. parahaemolyticus*_(AHPND)._ **(B)** ROS levels in shrimp hemocytes determined after *Pv*Kr-h1 knockdown and *V. parahaemolyticus*_(AHPND)_ challenge. Statistical analysis was performed by the unpaired Student’s t-test (ns, not significant; *p < 0.05; **p < 0.01).

After observing that antioxidant-related enzymes were dysregulated after *Pv*Kr-h1 knockdown followed by *V. parahaemolyticus*_(AHPND)_ challenge, we examined whether this could affect reactive oxygen species (ROS) production. When shrimp were depleted of *Pv*Kr-h1 followed by *V. parahaemolyticus*_(AHPND)_ challenge, the mRNA transcript levels of antioxidant-related genes in hemocytes, i.e., Cu-Zn SOD, GPX, and GST were significantly increased compared with control (**Figure 4A**). The level of ROS in shrimp hemocytes decreased significantly at 12 and 24 h in the *Pv*Kr-h1 knockdown followed by *V. parahaemolyticus*_(AHPND)_ challenge group compared the control (**Figure 4B**). These results suggest that *Pv*Kr-h1 mediate ROS production in shrimp in hemocytes by modulating the expression of antioxidant-related enzymes during *V. parahaemolyticus*_(AHPND)_ challenge.

### 3.5 *Pv*Kr-h1 modulates the entry of Relish in the nucleus during bacterial challenge to regulate antimicrobial peptides genes expression

**Figure 5:**
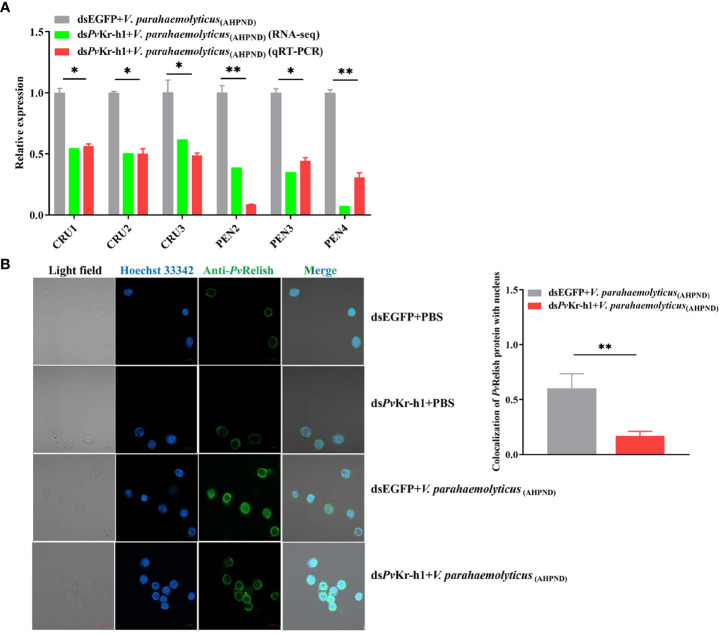
Modulation of antimicrobial peptide (AMP) genes and IMD/Relish pathway by Kr-h1 in *P. vannamei* hemocytes during *V. parahaemolyticus*_(AHPND)_ challenge. **(A)**. Expression of AMP genes after shrimp were injected with ds*Pv*Kr-h1 or dsEGFP (control) followed by *V. parahaemolyticus*_(AHPND)_, **(B)** Nuclei localization of *Pv*Relish in shrimp hemocytes after *Pv*Kr-h1 knockdown followed by *V. parahaemolyticus*_(AHPND)_ challenge. Statistical analysis was performed by the unpaired Student’s t-test (*p < 0.05; **p < 0.01).

During bacterial infections, the IMD pathway in shrimp is activated through a series of reaction cascades that cleave Relish to enable the entry of the Rel homology domain (RHD) to regulate AMPs expression (29). When shrimp were depleted of *Pv*Kr-h1, the mRNA transcript levels of AMPs genes, i.e., CRUs, PENs decreased significantly (**Figure 5A**), suggesting that *Pv*Kr-h1 could regulate the expression of AMPs, probably through the IMD pathway.

Given that during pathogenic bacteria infection in shrimp, Relish is reported to regulate the expression of AMPs by targeting their promoters (11) and that Relish is downstream of IMD in innate immune signaling, we determined whether *Pv*Kr-h1 knockdown affects the nuclei entry of *Pv*Relish. Using immunofluorescence analysis, we found that after *Pv*Kr-h1 knockdown followed by *V. parahaemolyticus*_(AHPND)_ challenge, the number of hemocytes with *Pv*Relish fluorescence signals in the nucleus was 75% less than the control (**Figure 5B**). These results indicate that *Pv*Kr-h1 could promote AMPs expression during *V. parahaemolyticus*_(AHPND)_ challenge through the IMD/Relish pathway.

## 4 Discussion

Many diseases have affected the shrimp aquaculture industry, especially AHPND, caused by a strain of *V. parahaemolyticus* (*V. parahaemolyticus*_(AHPND)_) that carries the PirA/PirB binary toxin plasmid. Extensive research has been undertaken to understand the pathogenesis and mechanism of *V. parahaemolyticus*_(AHPND)_ infection in shrimp, through which several factors have been implicated (1, 28, 30). However, the key transcription factors that modulate *V. parahaemolyticus*_(AHPND)_ infection and the molecular mechanisms involved are still elusive. Screening of our previously published *V. parahaemolyticus*_(AHPND)_ challenged shrimp hemocytes transcriptome data (GenBank accession number: PRJNA385392) revealed several dysregulated transcription factors, among which *Pv*Kr-h1 was the most expressed, suggesting that *Pv*Kr-h1 could be a crucial transcription factor in shrimp antimicrobial response to *V. parahaemolyticus*_(AHPND)_ infection (**Figure 1**).

Kruppel-like factors (KLFs) are transcription factors found widely in vertebrates and invertebrates and have evolutionarily conserved zinc finger domains that target DNA sequences (31). The krueppel homolog-1 (Kr-h1) gene was first identified in *Drosophila* in 1986 (18). While arthropodan Kr-h1 genes, such as those found in *Drosophila*, *Bombyx mori, T. castaneum*, etc., contain eight Znf-C2H2 domains, crustacean Kr-h1, as in *P. vannamei* (**Figure S1**), contain only seven Znf-C2H2 domains (19, 20). These differences in the domain structures between the arthropodan and crustacean Kr-h1 suggest that they might play different functions in these species. For instance, in *Drosophila*, Kr-h1 is important in regulating the antagonism of JH and ecdysone (21, 32, 33), whereas in crustaceans Kr-h1 is involved in methyl farnesoate (MF)-mediated vitellogenesis, and the JH/MF signaling pathway (20).

Many studies have revealed that the endocrine and immune systems are closely linked (34). For instance, in *Drosophila*, ecdysone is involved in the innate immune defense by regulating the production of AMPs, such as cecropin A1, attacin A, and deficiency A, through PGRP-LC, a key receptor in the IMD pathway (35). Thus, given the importance of Kr-h1 and its involvement in regulating key physiological processes in crustaceans, coupled with its implication in insects’ innate immune response, it is conceivable that Kr-h1 could also be involved in shrimp innate immune response, especially its induced expression during *V. parahaemolyticus*_(AHPND)_ challenge in shrimp. Indeed, besides a significant increase in *Pv*Kr-h1 mRNA transcript levels in shrimp hemocytes after *V. parahaemolyticus*_(AHPND)_ challenge, hemolymph total bacterial abundance and shrimp mortality increased significantly after *Pv*Kr-h1 knockdown followed by *V. parahaemolyticus*_(AHPND)_ challenge (**Figure 2**). This observation is consistent with a previous study where the expression of Kr-h1 gene in *T. castaneum* was significantly upregulated after *Paranosema whitei* infection (22). Moreover, our recent data revealed that juvenile hormone epoxide hydrolase (JHEH), an enzyme that breaks down juvenile hormone, which is also involved in the development and molting of insects, plays an essential role in shrimp survival during bacterial infection (36). Therefore, Kr-h1 is important in regulating key physiological processes in crustaceans and also plays an essential role in the innate immune response during AHPND in shrimp.

When transcriptome analysis of shrimp hemocytes after *Pv*Kr-h1 knockdown followed by *V. parahaemolyticus*_(AHPND)_ challenge was used to further explore how *Pv*Kr-h1 modulates shrimp innate immune response, 92 DEGs were found to be enriched immune system pathways after KEGG pathway enrichment analysis. Several antioxidant-related genes, i.e., Cu-Zn SOD, GST, and GPX, were among the significantly changed DEGs. Given that bacteria-induced stress can affect ROS production through the regulation of antioxidant enzymes (CAT, GPX, SOD, POD, and GST) (37), thus we explored how *Pv*Kr-h1 could modulate ROS production through the expression of antioxidant enzymes in shrimp hemocytes during *Pv*Kr-h1 knockdown followed by *V. parahaemolyticus*_(AHPND)_ challenge. Interestingly, the mRNA transcript levels of Cu-Zn SOD, GST, and GPX were significantly increased, whereas ROS levels were significantly decreased after *Pv*Kr-h1 knockdown followed by *V. parahaemolyticus*_(AHPND)_ challenge (**Figure 4**). Similar data have previously been reported in *Marsupenaeus japonicus*, where after shrimp were depleted of *Mj*DUOXs followed by *Vibrio anguillarium* infection, ROS levels decreased whereas bacterial abundance and shrimp mortality increased (38). Our data suggest that during *V. parahaemolyticus*_(AHPND)_ challenge, a decrease in *Pv*Kr-h1 expression reduces ROS production through induced antioxidant enzymes expression. The reduced ROS in hemocytes together with the increased bacterial load in hemolymph thereby promoted shrimp mortality.

During *V. parahaemolyticus*_(AHPND)_ infection *Pv*Kr-h1 also regulates the expression of AMP genes, such as CRUs and PENs, which is attenuated after *Pv*Kr-h1 knockdown (**Figure 5A**). This observation is corroborated by a previous study in *E. sinensis*, where the knockdown of EsDAZAP2 (azoospermia-associated protein 2) inhibited the expression of AMPs and increased bacterial abundance and crab mortality (39). In crustaceans, the expression of AMPs is mainly regulated by the Toll, IMD, and JAK/STAT pathways mediated by transcription factors, such as Dorsal, Relish, and STAT (40–42). Some previous studies have shown that Kr-h1 binds to KBS (Kr-h1 binding site) on the promoter of target genes to regulate their transcription (32, 43). Interestingly, two KBS sites were identified at -3579/-3574 bp and -3537/-3532 bp on the promoter of *Pv*Relish (**Figure S2**), suggesting that *Pv*Kr-h1 might be an upstream regulator of *Pv*Relish, which has been shown to modulate the expression of some antimicrobial peptides, such as PENs, during pathogen challenge (11). Here, we found that after *Pv*Kr-h1 knockdown, the nuclei translocation of *Pv*Relish from the cytoplasm is attenuated in shrimp hemocytes during *V. parahaemolyticus*_(AHPND)_ challenge (**Figure 5B**). This indicates that *Pv*Kr-h1 is important in the *Pv*Relish signaling pathway during the antimicrobial response in penaeid shrimp. A similar mechanism was found in the Chinese mitten crab *E. sinensis*, where EsDAZAP2 was reported to exert its immunological function by promoting the rate of Esdorsal nuclear translocation to increase the expression of AMPs (39).

**Figure 6:**
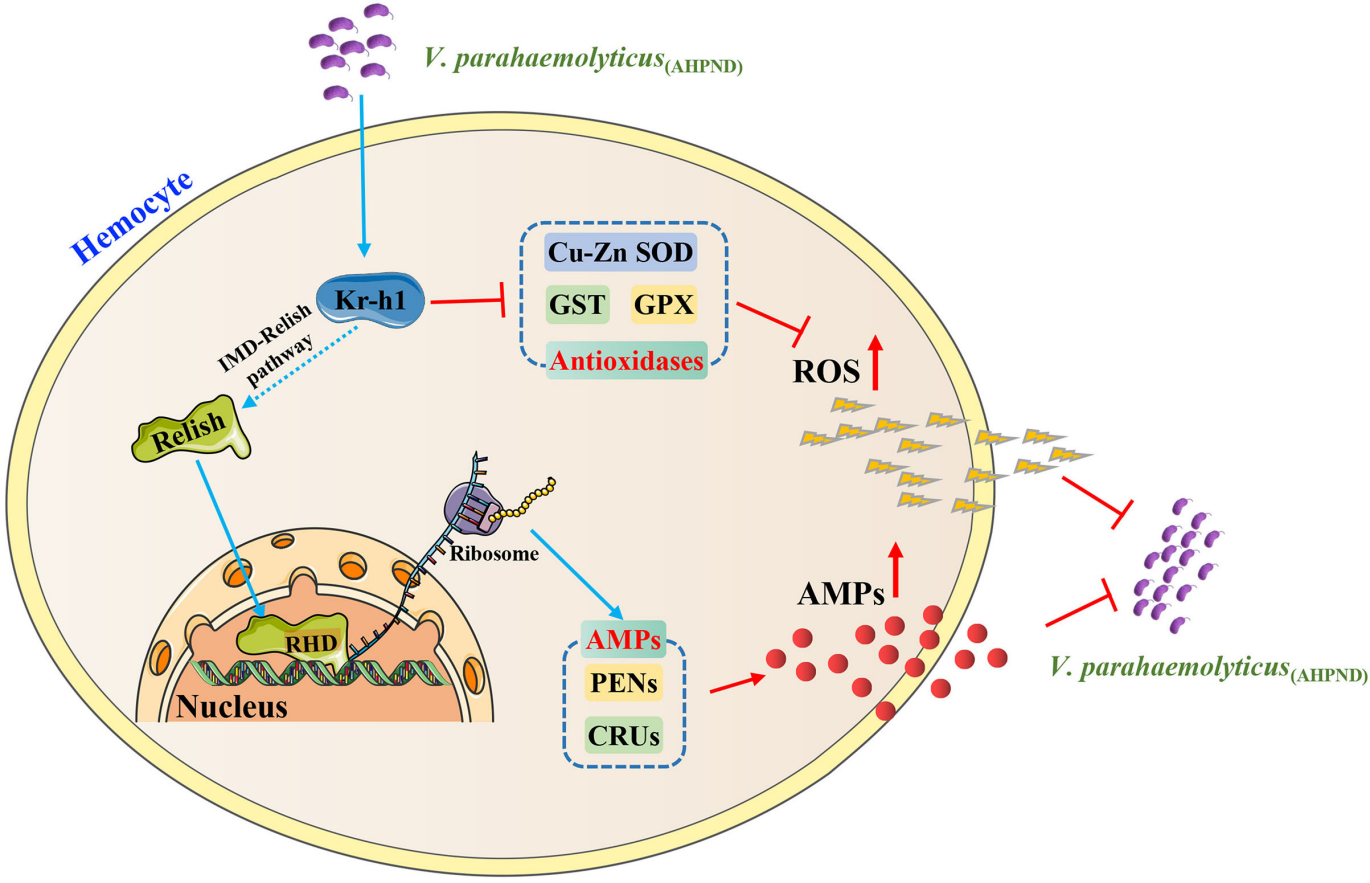
A proposed model of *Pv*Kr-h1 modulation of immune response during *V. parahaemolyticus*_(AHPND)_ challenge of *P. vannamei*. Kr-h1 is upregulated in shrimp hemocytes upon *V. parahaemolyticus*_(AHPND)_ infection, which activates the IMD/Relish pathway by the endoproteolytic cleavage of Relish to generate a DNA-binding Rel homology domain (RHD) that enters the nucleus to promote the expression of antimicrobial peptide genes (e.g., CRUs and PENs). Concurrently, high levels of Kr-h1 suppress the expression of antioxidant-related enzymes (e.g., GPX, GST, and Cu-Zn SOD), which enhances ROS production. The resulting high level of AMPs and ROS in hemocytes enhances bacterial clearance.

In conclusion, our current study revealed that *Pv*Kr-h1 regulates ROS production and AMPs expression through the IMD/Relish pathway in shrimp, indicating that *Pv*Kr-h1 plays an essential role in shrimp antimicrobial immune response to *V. parahaemolyticus*_(AHPND)_ infection (**Figure 6**). Future studies will aim to further the remaining unanswered questions, such as the specific mechanisms involved in the modulation of ROS production and antimicrobial peptides expression by *Pv*Kr-h1, by use of the chromatin immunoprecipitation assay, mutation of the KBS sites on *Pv*Kr-h1, and other related molecular methods..

## Data availability statement

The data presented in the study are deposited in the NCBI BioProject repository, accession number PRJNA875156.

## Ethics statement

The animal study was reviewed and approved by the Animal Research and Ethics Committees of Shantou University, Guangdong, China.

## Author contributions

YLZ and ZZ conceived and designed the experiments. YLZ, SJL and JA acquired funding. ZZ performed the experiments. YLZ, YZZ, and YC contributed reagents. ZHZ contributed analytic tools. YLZ and SJL supervised the work. ZZ and SJL wrote the original draft. JA, ZL, ZZ, YLZ and SKL reviewed and revised the manuscript. All authors have read and agreed to the published version of the manuscript. All authors contributed to the article and approved the submitted version.
