# Supplementary material for: Kruppel homolog 1 modulates ROS production and antimicrobial peptides expression in shrimp hemocytes during infection by the Vibrio parahaemolyticus strain that causes AHPND
Source: Front Immunol. 2023 Aug 29;14:1246181. doi: 10.3389/fimmu.2023.1246181 (PMC10497957; doi:10.3389/fimmu.2023.1246181)
Supplement: Supplementary file 1 [file DataSheet_1.doc]

**Table S1.** Upregulated diﬀerentially expressed immune-relate genes in the dsEGFP + *V. parahaemolyticus* _(AHPND)_ versus ds*Pv*Kr-h1 + *V. parahaemolyticus* _(AHPND)_ group

| No. | Gene_id | Regulate | Gene name | Gene description |
| --- | --- | --- | --- | --- |
| 1 | gene3267 | up | LOC113803123 | 6-phosphogluconate dehydrogenase |
| 2 | gene12182 | up | LOC113813021 | actin, cytoplasmic A3a-like |
| 3 | gene20654 | up | LOC113822454 | ADP-ribosylation factor-like |
| 4 | gene15035 | up | LOC113816222 | amidophosphoribosyltransferase-like |
| 5 | gene1136 | up | LOC113811998 | antileukoproteinase-like |
| 6 | gene94 | up | LOC113810045 | anti-lipopolysaccharide factor-like |
| 7 | gene9557 | up | LOC113810108 | anti-lipopolysaccharide factor-like |
| 8 | gene6918 | up | LOC113807192 | anti-lipopolysaccharide factor-like |
| 9 | gene10400 | up | LOC113811032 | anti-lipopolysaccharide factor-like |
| 10 | gene6917 | up | LOC113807191 | anti-lipopolysaccharide factor-like |
| 11 | gene20301 | up | LOC113822052 | astakine, transcript variant X2 |
| 12 | gene4373 | up | LOC113804338 | caspase-10-like |
| 13 | gene28362 | up | LOC113800243 | caspase-2-like |
| 14 | gene16345 | up | LOC113817651 | chymotrypsin A-like |
| 15 | gene5618 | up | LOC113805736 | chymotrypsin BII |
| 16 | gene8398 | up | LOC113808838 | CREB-binding protein-like |
| 17 | gene28868 | up | LOC113800797 | CTL-like protein 2, transcript variant X2 |
| 18 | gene7819 | up | LOC113808187 | C-type lectin |
| 19 | gene23136 | up | LOC113825161 | cytochrome c, transcript variant X1 |
| 20 | gene1753 | up | LOC113818864 | dual specificity protein phosphatase 7-like |
| 21 | gene25370 | up | LOC113827639 | dual specificity protein phosphatase 7-like |
| 22 | gene6044 | up | LOC113806222 | ficolin-1-like |
| 23 | gene6043 | up | LOC113806220 | ficolin-2-like |
| 24 | gene22015 | up | LOC113823946 | glutamine gamma-glutamyltransferase-like |
| 25 | gene24107 | up | LOC113826241 | glutamine gamma-glutamyltransferase-like |
| 26 | gene15468 | up | LOC113816686 | glutathione peroxidase-like |
| 27 | gene1273 | up | LOC113813522 | glutathione S-transferase 1-like |
| 28 | gene18210 | up | LOC113819746 | hemolymph clottable protein-like |
| 29 | gene5598 | up | LOC113805724 | histone acetyltransferase KAT6A-like |
| 30 | gene22065 | up | LOC113823990 | interferon alpha-inducible protein 27-like protein 2 |
| 31 | gene22064 | up | LOC113823997 | interferon alpha-inducible protein 27-like protein 2B |
| 32 | gene25441 | up | LOC113827723 | interferon alpha-inducible protein 27-like protein 2B |
| 33 | gene7630 | up | LOC113807973 | interferon regulatory factor 2-binding protein 2-B-like |
| 34 | gene25571 | up | LOC113827868 | legumain-like |
| 35 | gene22562 | up | LOC113824530 | leucine-rich repeat-containing protein |
| 36 | gene24085 | up | LOC113826216 | lysosome membrane protein 2-like |
| 37 | gene3735 | up | LOC113803638 | mucin-2-like |
| 38 | gene23745 | up | LOC113825840 | mucin-5AC-like |
| 39 | gene11825 | up | LOC113812615 | mucin-5AC-like |
| 40 | gene23772 | up | LOC113825867 | myosin heavy chain, cardiac muscle isoform-like |
| 41 | gene23773 | up | LOC113825871 | myosin heavy chain, cardiac muscle isoform-like |
| 42 | gene15993 | up | LOC113817273 | myosin heavy chain-like |
| 43 | gene19217 | up | LOC113820858 | myosin-IB-like |
| 44 | gene21747 | up | LOC113823636 | NF-kappa-B inhibitor cactus-like |
| 45 | gene1872 | up | LOC113820005 | perlucin-like protein |
| 46 | gene10729 | up | LOC113811390 | peroxiredoxin-like 2A |
| 47 | gene21414 | up | LOC113823275 | phenoloxidase-activating factor 1-like |
| 48 | gene8632 | up | LOC113809076 | phenoloxidase-activating factor 2-like |
| 49 | gene22342 | up | LOC113824294 | phenoloxidase-activating factor 3-like |
| 50 | gene26074 | up | LOC113828431 | phenoloxidase-activating factor 3-like |
| 51 | gene9982 | up | LOC113810575 | protein spaetzle 5-like, transcript variant X1 |
| 52 | gene28504 | up | LOC113800366 | serine protease inhibitor 88Ea-like |
| 53 | gene8995 | up | LOC113809456 | serine/threonine-protein kinase H1 homolog |
| 54 | gene24389 | up | LOC113826536 | serine/threonine-protein kinase pim-3-like |
| 55 | gene16653 | up | LOC113817999 | superoxide dismutase [Cu-Zn]-like |
| 56 | gene9129 | up | LOC113809633 | superoxide dismutase [Cu-Zn]-like |
| 57 | gene22932 | up | LOC113824943 | techylectin-5A-like |
| 58 | gene30477 | up | LOC113802550 | triosephosphate isomerase B-like |
| 59 | gene30478 | up | LOC113802551 | triosephosphate isomerase-like |
| 60 | gene12862 | up | LOC113813774 | trypsin-1-like |
| 61 | gene26267 | up | LOC113828658 | trypsin-1-like |

**Table S2.** Downregulated diﬀerentially expressed immune-relate genes in the dsEGFP + *V. parahaemolyticus* _(AHPND)_ versus ds*Pv*Kr-h1 + *V. parahaemolyticus* _(AHPND)_ group

| No. | Gene_id | Regulate | Gene name | Gene description |
| --- | --- | --- | --- | --- |
| 1 | gene20655 | down | LOC113822447 | ADP-ribosylation factor-like |
| 2 | gene26650 | down | LOC113829072 | alpha-2-macroglobulin-like |
| 3 | gene11474 | down | LOC113812204 | anti-lipopolysaccharide factor-like |
| 4 | gene28308 | down | LOC113800177 | chymotrypsin-like elastase family member 2A |
| 5 | gene29798 | down | LOC113801825 | crustin 1 |
| 6 | gene18624 | down | [LOC113820206](https://www.ncbi.nlm.nih.gov/gene/?term=113820206) | crustin 3 |
| 7 | gene15085 | down | LOC113816267 | crustin-like protein |
| 8 | gene11455 | down | LOC113812219 | C-type lectin |
| 9 | gene23650 | down | LOC113825732 | cuticle protein 7-like |
| 10 | gene17667 | down | LOC113819135 | cuticle protein 8-like |
| 11 | gene25495 | down | LOC113827783 | cytochrome c oxidase |
| 12 | gene5902 | down | LOC113806070 | dual specificity mitogen-activated protein kinase kinase 6-like |
| 13 | gene23764 | down | LOC113825861 | fibrinogen C domain-containing protein |
| 14 | gene6796 | down | LOC113807056 | kelch-like protein 5, transcript variant X1 |
| 15 | gene20771 | down | LOC113822590 | Krueppel homolog 1-like |
| 16 | gene25569 | down | LOC113827865 | legumain-like, transcript variant X2 |
| 17 | gene22804 | down | LOC113824801 | myosin heavy chain |
| 18 | gene16755 | down | LOC113818114 | NF-kappa-B-repressing factor-like |
| 19 | gene8548 | down | LOC113808998 | penaeidin-2b-like |
| 20 | gene8547 | down | LOC113808997 | penaeidin-3a-like |
| 21 | gene8546 | down | LOC113808996 | penaeidin-4 |
| 22 | gene11625 | down | LOC113812399 | programmed cell death protein 4-like |
| 23 | gene11011 | down | LOC113811712 | proliferating cell nuclear antigen-like |
| 24 | gene22382 | down | LOC113824360 | protein toll-like |
| 25 | gene23252 | down | LOC113825263 | serine protease inhibitor 88Ea-like |
| 26 | gene14947 | down | LOC113816123 | triosephosphate isomerase B-like |
| 27 | gene29052 | down | LOC113801022 | triosephosphate isomerase B-like |
| 28 | gene29056 | down | LOC113801019 | triosephosphate isomerase-like |
| 29 | gene29067 | down | LOC113801014 | triosephosphate isomerase-like |
| 30 | gene29048 | down | LOC113801005 | triosephosphate isomerase-like |
| 31 | gene16471 | down | LOC113817800 | tubulin alpha-2/alpha-4 chain-like |

**A.**

**B.**


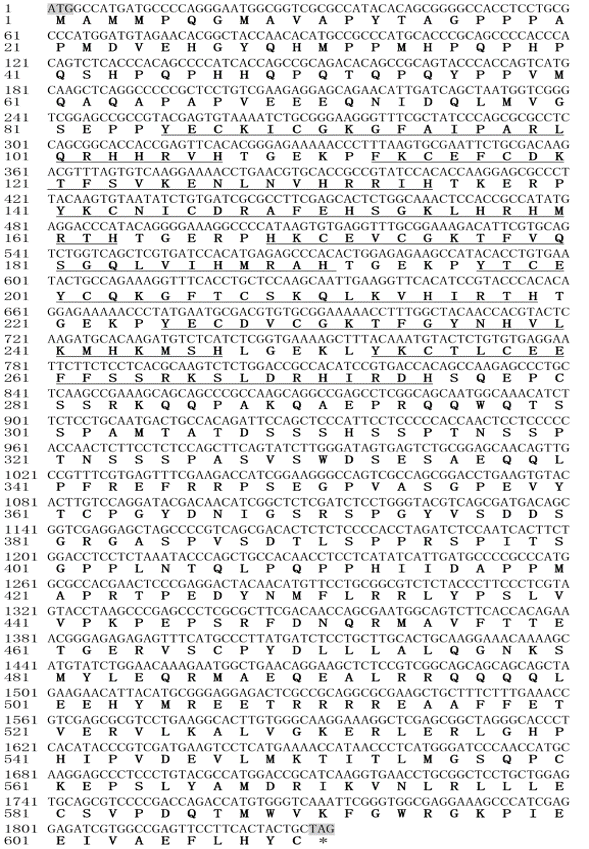

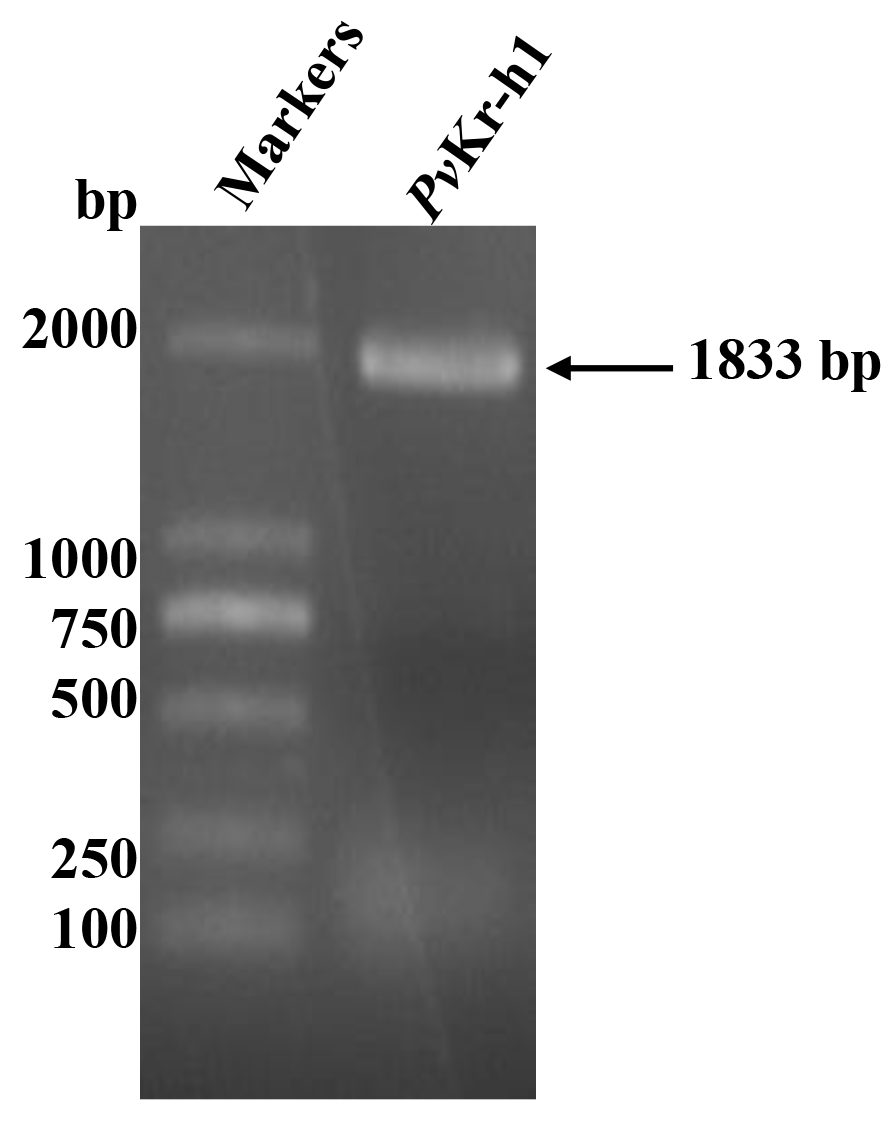


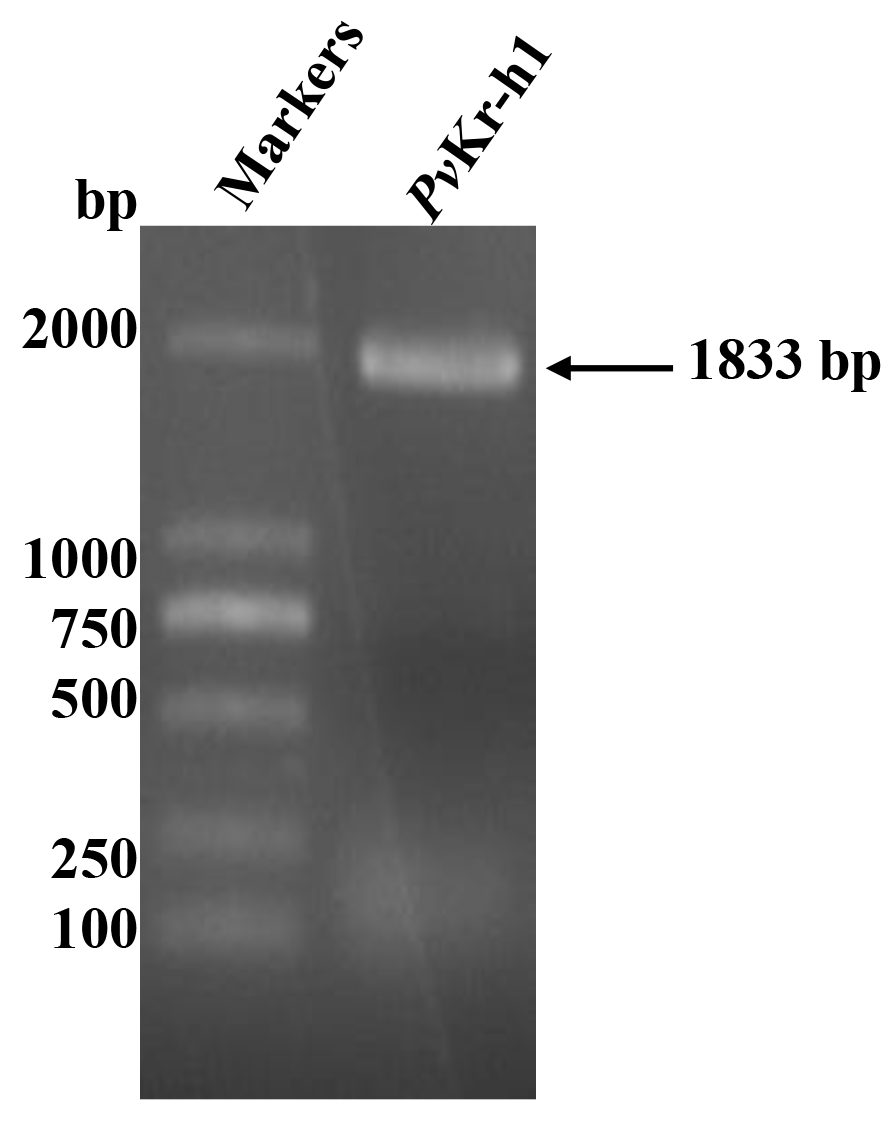

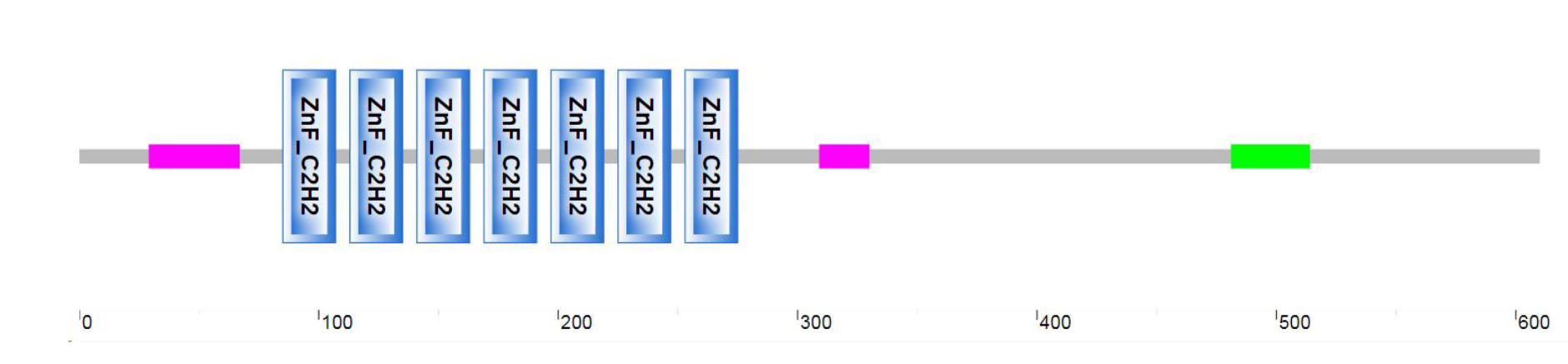


**C.**

**D.**

**E.**


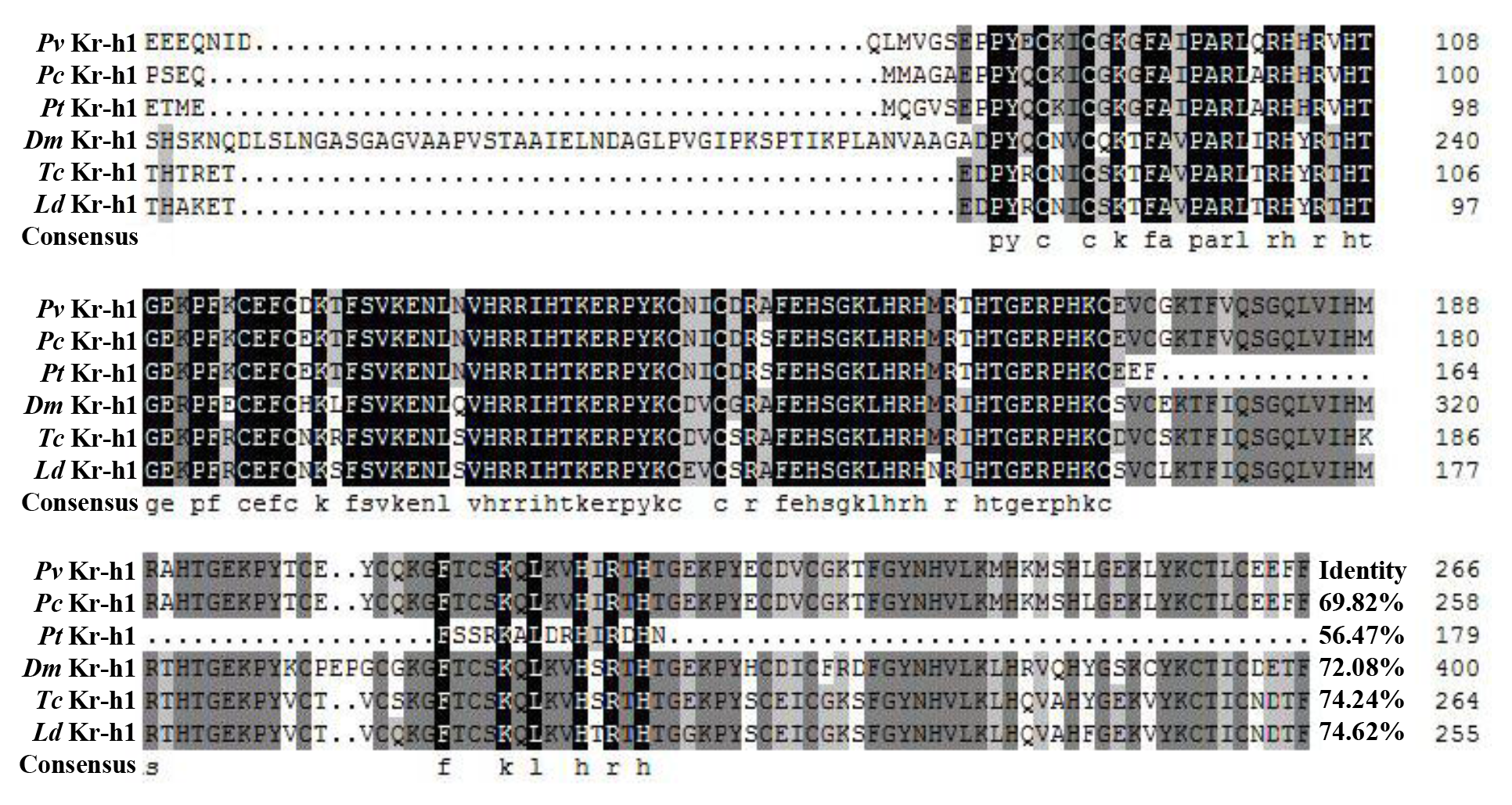


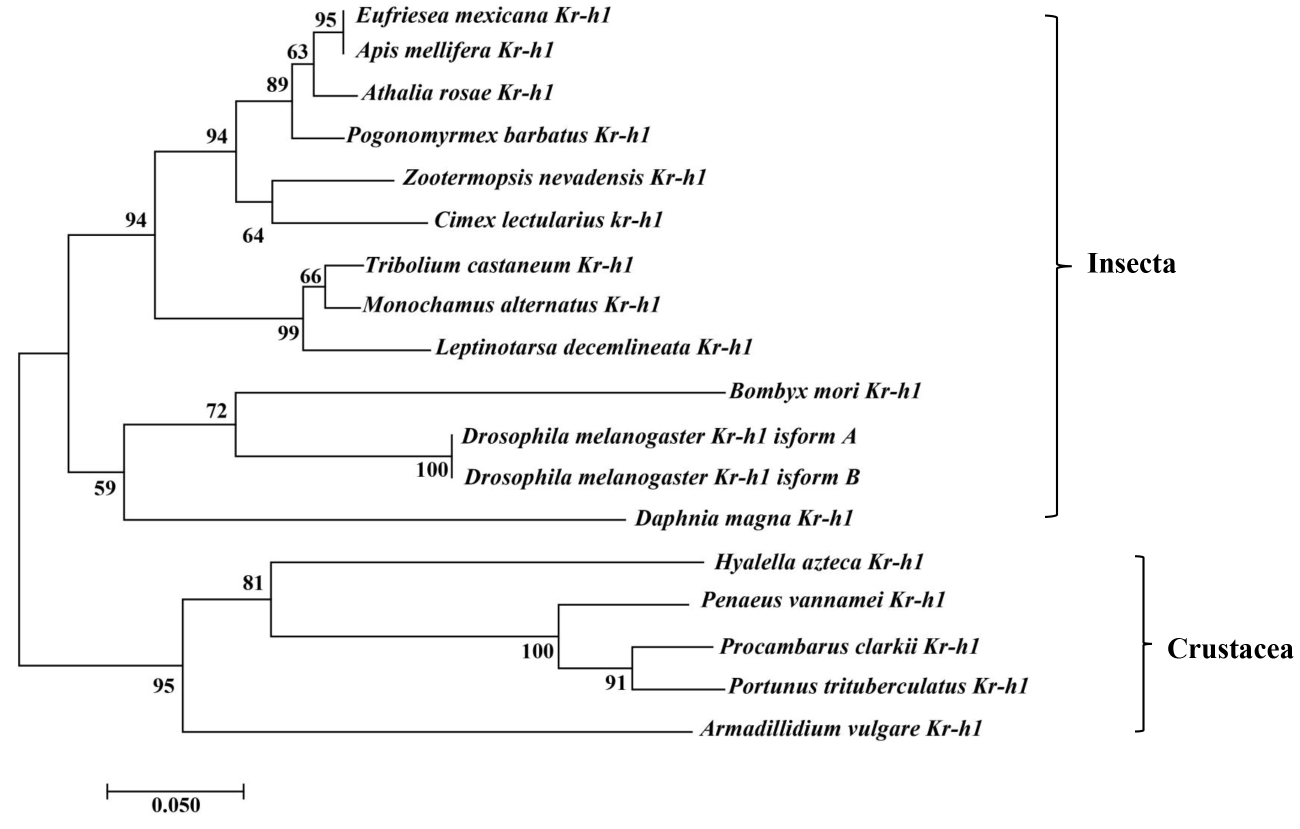


**Fig. S1.** *Pv*Kr-h1 is an important member of the Krupple family. (A) Cloning of the full-length cDNA of *Pv*Kr-h1 PCR amplification. (B). Full-length open reading frame (ORF) nucleotide sequence of *Pv*Kr-h1 (NW_020870886.1) and deduced amino acid sequence. (C) Predicted protein functional domain of *Pv*JHEH1. (D) Multiple sequence alignment of *Pv*Kr-h1 and JHEH from other species. All sequences were retrieved from NCBI databases, including *Penaeus vannamei* Kr-h1 (XP_027230918.1), *Procambarus clarkia* (QIA97604.1), *Portunus trituberculatus* (MPC40102.1), *Drosophila melanogaster* (NP_477467.1), *Tribolium castaneum* (EFA03084.2), and *Leptinotarsa decemlineata* (AGT57869.1). (E). Phylogenetic tree analysis of PvKr-h1 with other species Kr-h1. All sequences were retrieved from NCBI databases, including *Eufriesea Mexicana* (XP_017758246.1), *Apis mellifera* (BAL04728.1), *Athalia rosae* (XP_012267681.1), *Pogonomyrmex barbatus* (XP_011632498.1), *Zootermopsis nevadensis* (BAR92641.1), *Cimex lectularius* (XP_014253450.1), *Tribolium castaneum* (EFA03084.2), *Monochamus alternatus* (ANW09587.1), *Leptinotarsa decemlineata* (AGT57869.1), *Bombyx mori* (NP_001171332.1), *Drosophila melanogaster* (NP_477467.1), *Drosophila melanogaster* (NP_477466.1), *Daphnia magna* (XP_032780555.1), *Hyalella azteca* (XP_018027646.1), *Penaeus vannamei* (XP_027230918.1), *Procambarus clarkii* (QZA97604.1), *Portunus trituberculatus* (MPC40102.1), *Armadillidium vulgare* (RXG67945.1).


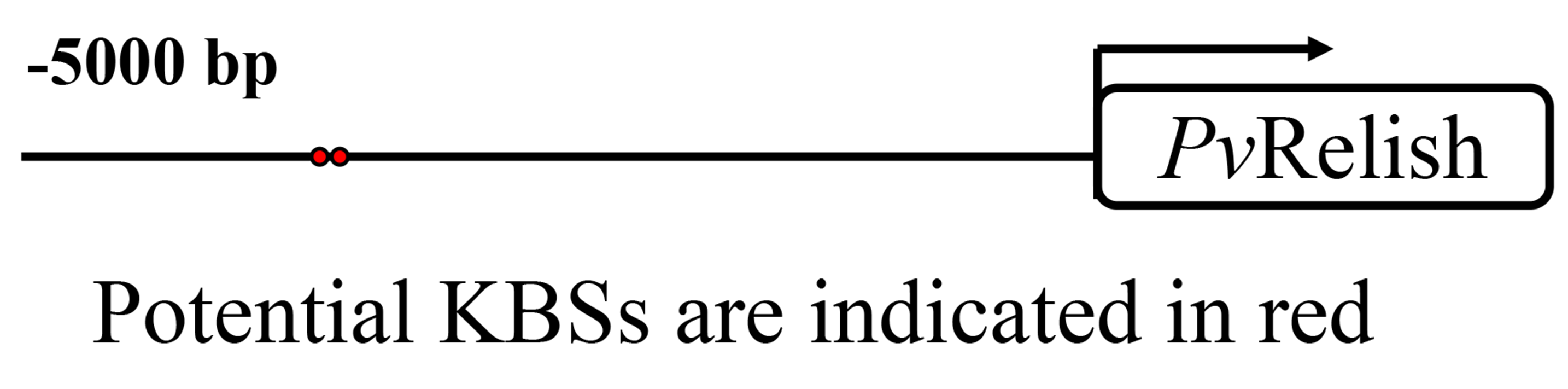


**Fig. S2.** Predicted potential KBS binding sites on *Pv*Relish promoter
